# Association of a novel nutritional index with cognitive impairment in middle-aged and elderly Chinese adults: a cross-sectional analysis from the China Health and Retirement Longitudinal Study

**DOI:** 10.3389/fnut.2025.1486917

**Published:** 2025-02-03

**Authors:** Guotao Liu, Jianyuan Zhang

**Affiliations:** ^1^Department of Health Care, Qilu Hospital, Cheeloo College of Medicine, Shandong University, Qingdao, China; ^2^Department of Neurology, Qilu Hospital, Cheeloo College of Medicine, Shandong University, Qingdao, China

**Keywords:** nutritional index, TCBI, cognitive impairment, CHARLS, cross-sectional study

## Abstract

**Purpose:**

The triglyceride-cholesterol-body weight index (TCBI), a novel and easily computable nutritional index, incorporates serum triglyceride (TG), total cholesterol (TC), and body weight (BW). This study explored the association between TCBI and cognitive impairment in middle-aged and elderly Chinese populations.

**Patients and methods:**

This cross-sectional study employed data from the China Health and Retirement Longitudinal Study (CHARLS) baseline survey, including 7,145 participants. TCBI was calculated as TG (mg/dL) × TC (mg/dL) × BW (kg)/1,000. Cognitive function was assessed based on mental status and episodic memory, with a total score below 11 indicating cognitive impairment. The relationship between TCBI and cognitive impairment was examined using multiple logistic regression, smooth curve fitting, and subgroup analyses.

**Results:**

After full adjustment, each 1-unit increase in log-transformed TCBI (Lg TCBI) was associated with a 29.7% reduction in cognitive impairment risk [odds ratio (OR) = 0.703, 95% confidence interval (CI): 0.529–0.933; *p* = 0.015]. When Lg TCBI was categorized into quartiles, the Q2, Q3, and Q4 groups exhibited a reduced risk of cognitive impairment by 19.9, 16.3, and 22.9%, respectively (*p* for trend = 0.043), compared to the Q1 group. Smooth curve fitting revealed a consistent decrease in cognitive impairment risk with higher Lg TCBI levels. Subgroup analysis indicated that the association was stronger among participants aged ≥60 years (OR = 0.655, 95% CI: 0.438–0.979), non-current drinkers (OR = 0.643, 95% CI: 0.451–0.917), and those who engaged in socializing (OR = 0.568, 95% CI: 0.371–0.871).

**Conclusion:**

TCBI was significantly and negatively associated with cognitive impairment in Chinese middle-aged and elderly individuals, with the effect more pronounced in those aged ≥60 years, non-current drinkers, and socially active participants.

## Introduction

Cognitive impairment comprises various cognitive dysfunctions, ranging from mild cognitive impairment (MCI) to dementia. An epidemiological survey conducted in 2020 reported that approximately 38.77 million individuals aged ≥60 years in China had MCI, while 15.07 million were affected by dementia (1). In aging populations, cognitive impairment imposes significant social and economic burdens on global public health systems. Therefore, early identification and mitigation of risk factors associated with cognitive impairment are crucial.

Cognitive impairment is linked to several risk factors, including advanced age, female gender, family history of dementia, living alone, rural residence, low educational attainment, smoking, hyperlipidemia, and cerebrovascular disease (1–5). Recent studies increasingly highlight malnutrition as a significant risk factor for cognitive impairment with nutrition playing a crucial role in preventing cognitive decline (6–9). However, existing nutritional assessment tools, such as the Prognostic Nutritional Index (PNI) (10), Controlling Nutritional Status (CONUT) (11), and the Geriatric Nutritional Risk Index (GNRI) (12), have not been extensively adopted in clinical practice owing to their complexity. In response to this gap, Doi et al. (13) proposed a new, easily calculable index known as triglycerides (TG), total cholesterol (TC), and body weight (BW) index (TCBI). The TCBI is calculated using the following formula:


$$TG\;\left(\mathrm{mg}/\mathrm{dL}\right)\times TC\;\left(\mathrm{mg}/\mathrm{dL}\right)\times BW\;\left(\mathrm{kg}\right)/1,000.$$

Previous studies have indicated that TCBI acts as a prognostic indicator for coronary heart disease (13), critical illness requiring mechanical circulatory support (MCS) devices (14), and heart failure (15). TCBI also negatively correlates with the incidence of stroke (16) and stroke-associated pneumonia (SAP) (17). However, the relationship between TCBI and cognitive function remains unexplored. We hypothesize that higher TCBI levels may reduce the risk of cognitive impairment. Therefore, this study systematically evaluated the association between TCBI and cognitive impairment. Additionally, we explored potential moderating factors that may influence this association.

## Materials and methods

### Study population

This study used data from the China Health and Retirement Longitudinal Study (CHARLS).^1^ CHARLS collects high-quality micro-level longitudinal survey data on households and individuals aged ≥45 years in China, by focusing on the health status of the elderly population. The baseline survey, conducted in 2011, included 17,708 participants across 150 regions and 450 villages or urban communities nationwide, providing a representative snapshot of the middle-aged and elderly population in China (18). The research employed face-to-face computer-assisted personal interview, with follow-up surveys conducted every 2 years. Data collected encompassed demographic information, health status, health behaviors, social participation, and pension insurance. This study was approved by the Biomedical Ethics Committee of Peking University (IRB00001052-11015), and all procedures adhered to the Declaration of Helsinki. Informed consent was obtained from all participants.

**Figure 1:**
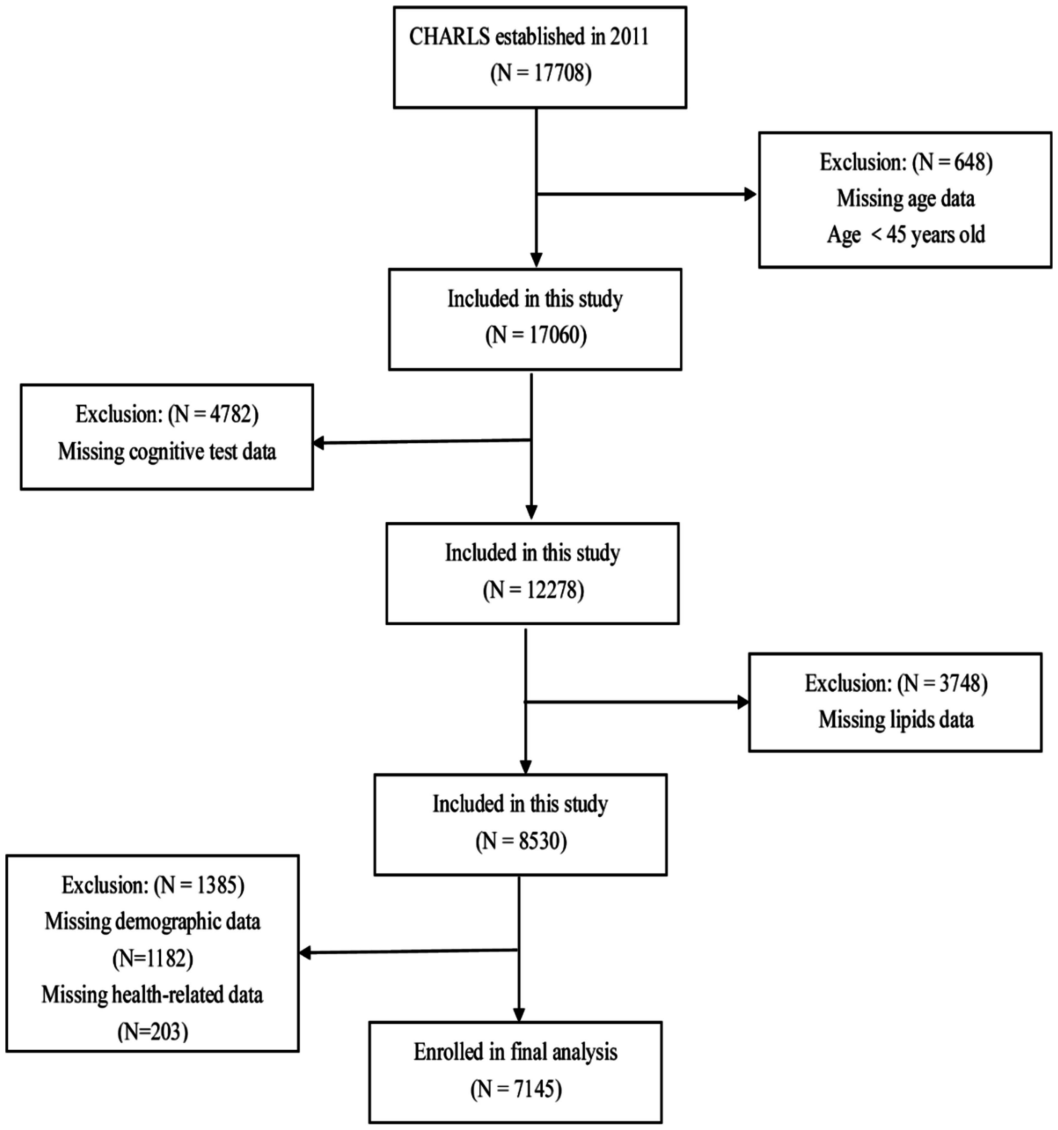
Participant selection process flowchart.

This study conducted a cross-sectional analysis of baseline data from CHARLS 2011. Participants were excluded based on the following criteria: age <45, absence of lipid profile data, lack of cognitive assessment data, or missing other demographic or health-related information. Ultimately, 7,145 participants were included (Figure 1).

### Assessment of cognitive function

Cognitive function of the CHARLS study population was evaluated using the methodology of the American Health and Retirement Study (HRS) (19). This assessment encompassed two domains: mental status and episodic memory. Mental status was evaluated through data recognition, calculation, and drawing ability tests, with a maximum score of 11 points. Episodic memory was assessed using word recall tasks in which interviewers read a list of 10 words to participants, who were then asked to recall as many words as possible. After a 5-min interval, participants were required to recall the words again. Each correctly recalled word was scored as 1 point, with a maximum score of 20 points for episodic memory. The total cognitive assessment score was 31. Consistent with previous studies (19, 20), cognitive impairment was defined as a score <11.

### Definition of TCBI

Venous blood samples were collected from participants in the CHARLS study, subsequently isolated, and transported to the Chinese Center for Disease Control and Prevention (China CDC) in Beijing. The samples were stored at −80°C and later examined at the Clinical Laboratory Center of Capital Medical University (CMU). Lipid profile tests, including TC, low-density lipoprotein cholesterol (LDL-C), high-density lipoprotein cholesterol (HDL-C), and TG levels, were performed using enzymatic colorimetric methods. The TCBI formula is as follows: TCBI = TG (mg/dL) × TC (mg/dL) × BW (kg)/1,000. High-sensitivity C-reactive protein (hsCRP) levels were measured using an immunoturbidimetric assay.

### Covariates

Demographic and health-related factors were used as covariates. Demographic factors included age, sex, residence, education level, marital status, and employment status. Health-related factors included body mass index (BMI), systolic blood pressure (SBP), diastolic blood pressure (DBP), stroke, diabetes, hypertension, dyslipidemia, depressive symptoms, social engagement, and current smoking and drinking habits. BMI was calculated as weight divided by height squared (kg/m^2^).

In CHARLS, hypertension was defined as a self-reported diagnosis of hypertension or blood pressure measurements indicating DBP ≥90 mmHg or SBP ≥140 mmHg. Diabetes was defined as self-reported physician-diagnosed diabetes or blood test results showing fasting plasma glucose (FPG) ≥126 mg/dL or glycated hemoglobin (HbA1c) ≥6.5%. Other chronic diseases were self-reported by participants. The CHARLS survey utilized the Center for Epidemiological Studies Depression (CESD-10) scale to assess depressive risk among older adults, with a score of ≥10 indicating high depressive symptoms.

In the 2011 CHARLS survey, there were a total of 11 different types of social participation. These activities include socializing with friends; community activity room (mahjong, chess, card games, etc.); dancing, or exercising in parks, or other venues; participating in club activities; attending school or training courses; providing assistance to relatives, friends, or neighbors who do not live together; caring for patients or individuals with disabilities who do not live together; engaging in volunteer activities or charity events; stock trading; going online; and other activities. Social engagement was defined as participation in at least one of the above 11 activities in the past month.

### Statistical analysis

Continuous variables were reported as mean ± standard deviation (normal distribution) or median with interquartile range (for skewed distribution). Categorical variables were presented as frequencies with percentages. Group comparisons of continuous variables were performed using one-way ANOVA or the Kruskal–Wallis *H* test. Categorical variables were compared using the chi-square test or the Kruskal–Wallis *H* test. Owing to the skewed distributions of the TCBI, it was transformed using the logarithm (log) to achieve normally. The association between clinical characteristics and cognitive impairment was assessed using a univariate logistic regression model. A multivariate logistic regression model, adjusted for age, sex, residence, education, marital status, retirement, current smoking, current drinking, socializing, hypertension, diabetes, dyslipidemia, stroke, depression symptoms, SBP, DBP, BMI, FPG, LDL-C, HDL-C, and hsCRP, was used to explore the relationship between TCBI and cognitive impairment.

Additionally, a detailed exploration of the association between TCBI and cognitive impairment was conducted using a generalized additive model with fitted smoothing curves. Stratified multivariate logistic regression models were used for subgroup analyses, according to age (45–60 and ≥60 years), sex, BMI (<24 and ≥24 kg/m^2^), residence (city and rural), education (illiteracy, primary school, middle school and high school and above), marital status (married and cohabiting and others), retirement, current smoking, current drinking, socializing, hypertension, diabetes, dyslipidemia, stroke, and depressive symptoms. The likelihood ratio test was used to examine interactions between subgroups. Finally, we conducted a sensitivity analysis among participants with normal blood lipid levels. All data analyses were performed using Statistical Product and Service Solutions software (SPSS, version 26.0) and EmpowerStats (http://www.empowerstats.com, X&Y Solutions, Inc., Boston, MA). A *p*-value of <0.05 (two-tailed) was considered statistically significant.

## Results

### Clinical characteristics based on TCBI quartiles

A flowchart of the participants is shown in Figure 1. A total of 7,145 participants were included in the study. The baseline characteristics of the participants are shown in Table 1. The average age of the participants was 58.86 ± 8.93 years, with 50.7% being males. The participants were divided into four groups based on the TCBI quartiles: Q1 (<749), Q2 (749–1,185), Q3 (1,185–1,938), and Q4 (≥1,938). No significant difference was observed in the proportion of stroke cases among the different TCBI groups (*p* > 0.05). In the high TCBI group (Q4), in contrast to the other three groups (Q1–Q3), participants had a higher proportion of females, rural residents, low education, married or cohabiting, socially engaged, non-retired, non-current smokers, non-current drinkers, and higher rates of hypertension, diabetes mellitus, dyslipidemia, and depressive symptoms. They also had higher levels of SBP, DBP, BMI, body weight, FPG, TC, TG, LDL-C, and hsCRP, but lower levels of age and HDL-C (*p* < 0.05). Additionally, the Q4 group had higher cognitive scores and a lower proportion of cognitively impaired participants (*p* < 0.05) than the Q1–Q3 groups.

**Table 1:** The characteristics of participants according to quartiles of TCBI.

Characteristics	TCBI quartiles				*p*-value
	Q1	Q2	Q3	Q4	
*N*	1,786	1,787	1,785	1,787	
Age (years)	59.86 ± 9.56	59.00 ± 9.02	58.84 ± 8.77	57.73 ± 8.20	<0.001
**Sex**					<0.001
Male	991 (55.49%)	930 (52.04%)	847 (47.45%)	855 (47.85%)	
Female	795 (44.51%)	857 (47.96%)	938 (52.55%)	932 (52.15%)	
**Residence**					<0.001
City	536 (30.01%)	628 (35.14%)	693 (38.82%)	811 (45.38%)	
Rural	1,250 (69.99%)	1,159 (64.86%)	1,092 (61.18%)	976 (54.62%)	
**Education**					<0.001
Below primary school	829 (46.42%)	724 (40.51%)	737 (41.29%)	689 (38.56%)	
Primary school	422 (23.63%)	446 (24.96%)	405 (22.69%)	432 (24.17%)	
Middle school	377 (21.11%)	400 (22.38%)	413 (23.14%)	443 (24.79%)	
High school and above	158 (8.85%)	217 (12.14%)	230 (12.89%)	223 (12.48%)	
**Marital status**					0.006
Married and cohabiting	1,557 (87.18%)	1,595 (89.26%)	1,604 (89.86%)	1,620 (90.60%)	
Others	229 (12.82%)	192 (10.74%)	181 (10.14%)	167 (9.35%)	
**Retirement**					<0.001
No	1,633 (91.43%)	1,593 (89.14%)	1,542 (86.39%)	1,507 (84.33%)	
Yes	153 (8.57%)	194 (10.86%)	243 (13.61%)	280 (15.67%)	
**Current smoking**					<0.001
No	1,121 (62.77%)	1,173 (65.64%)	1,260 (70.59%)	1,277 (71.46%)	
Yes	664 (37.18%)	614 (34.36%)	525 (29.41%)	510 (28.54%)	
**Current drinking**					0.008
No	1,108 (62.04%)	1,146 (64.13%)	1,198 (67.11%)	1,180 (66.03%)	
Yes	678 (37.96%)	641 (35.87%)	587 (32.89%)	607 (33.97%)	
**Socializing**					<0.001
No	943 (52.80%)	855 (47.85%)	791 (44.31%)	751 (42.03%)	
Yes	843 (47.20%)	932 (52.15%)	994 (55.69%)	1,036 (57.97%)	
**Hypertension**					<0.001
No	1,117 (62.54%)	1,020 (57.08%)	880 (49.30%)	724 (40.51%)	
Yes	669 (37.46%)	767 (42.92%)	905 (50.70%)	1,063 (59.49%)	
**Diabetes**					<0.001
No	1,634 (91.49%)	1,586 (88.75%)	1,529 (85.66%)	1,332 (74.54%)	
Yes	152 (8.51%)	201 (11.25%)	256 (14.34%)	455 (25.46%)	
**Dyslipidemia**					<0.001
No	1,701 (95.24%)	1,648 (92.22%)	1,605 (89.92%)	1,456 (81.48%)	
Yes	85 (4.76%)	139 (7.78%)	180 (10.08%)	331 (18.52%)	
**Stroke**					0.267
No	1,754 (98.21%)	1,748 (97.82%)	1,737 (97.31%)	1,741 (97.43%)	
Yes	32 (1.79%)	39 (2.18%)	48 (2.69%)	46 (2.57%)	
**Depressive symptoms**					<0.001
No	1,084 (60.69%)	1,139 (63.74%)	1,168 (65.43%)	1,222 (68.38%)	
Yes	702 (39.31%)	648 (36.26%)	617 (34.57%)	565 (31.62%)	
SBP (mmHg)	125.54 ± 21.20	127.56 ± 20.80	130.49 ± 21.19	133.56 ± 20.61	<0.001
DBP (mmHg)	72.57 ± 11.78	74.52 ± 11.91	76.20 ± 12.07	78.78 ± 11.98	<0.001
BMI (kg/m^2^)	21.35 ± 2.86	22.81 ± 3.16	24.32 ± 3.54	26.03 ± 4.07	<0.001
Body weight (kg)	53.14 ± 8.83	57.49 ± 9.44	61.59 ± 10.60	66.71 ± 12.14	<0.001
FPG (mg/dL)	98.64 (91.21, 106.92)	100.44 (93.78, 109.8)	102.96 (95.58, 113.04)	109.08 (99.54, 126.00)	<0.001
TC (mg/dL)	169.12 ± 29.51	187.45 ± 31.32	199.30 ± 32.16	218.35 ± 38.88	<0.001
TG (mg/dL)	61.95 (52.21, 72.57)	89.39 (77.88, 103.55)	125.67 (107.09, 145.14)	207.98 (167.27, 278.78)	<0.001
LDL-C (mg/dL)	99.68 ± 25.29	116.12 ± 28.53	126.13 ± 31.73	124.46 ± 43.77	<0.001
HDL-C (mg/dL)	58.82 ± 15.06	54.72 ± 14.75	48.72 ± 12.73	41.61 ± 11.90	<0.001
hsCRP (mg/L)	0.80 (0.45, 1.83)	0.88 (0.49, 1.94)	1.08 (0.59, 2.14)	1.32 (0.74, 2.62)	<0.001
Cognitive score	14.84 ± 4.90	15.54 ± 4.92	15.61 ± 4.80	16.19 ± 4.74	<0.001
**Cognitive impairment**					<0.001
No	1,326 (74.24%)	1,436 (80.36%)	1,438 (80.56%)	1,495 (83.66%)	
Yes	460 (25.76%)	351 (19.64%)	347 (19.44%)	292 (16.34%)	

BMI, body mass index; DBP, diastolic blood pressure; FPG, fasting plasma glucose; HDL-C, high-density lipoprotein cholesterol; hsCRP, high-sensitivity C-reactive protein; LDL-C, low-density lipoprotein cholesterol; SBP, systolic blood pressure; TC, total cholesterol; TCBI, triglycerides, total cholesterol, and body weight index; TG, triglyceride. TCBI was classified as Q1 (<749), Q2 (749–1,185), Q3 (1,185–1,938), and Q4 (≥1,938).

### Association between clinical characteristics and cognitive impairment

As shown in Table 2, univariate logistic regression analysis indicated that age, living in rural areas, hypertension, depressive symptoms, SBP, HDL-C levels, and hsCRP levels were positively associated with cognitive impairment (*p* < 0.05). Conversely, male sex, high education level, married and cohabiting, retired, current smoking, current drinking, social activity, dyslipidemia, BMI, TG, and TCBI were negatively associated with cognitive impairment (*p* < 0.05). However, diabetes, stroke, DBP, and FPG, TC, while LDL-C levels were not associated with cognitive impairment (*p* > 0.05).

**Table 2:** The correlation between clinical characteristics and cognitive impairment.

Characteristics	Statistics	OR (95% CI)	*p*-value
Age (years)	58.86 ± 8.93	1.063 (1.056, 1.070)	<0.001
Sex			
Female	3,522 (49.29%)	Reference	
Male	3,623 (50.71%)	0.535 (0.475, 0.602)	<0.001
Residence			
City	2,668 (37.34%)	Reference	
Rural	4,477 (62.66%)	2.009 (1.765, 2.288)	<0.001
Education			
Below primary school	2,979 (41.69%)	Reference	
Primary school	1,705 (23.86%)	0.249 (0.212, 0.292)	<0.001
Middle school	1,633 (22.86%)	0.100 (0.080, 0.125)	<0.001
High school and above	828 (11.59%)	0.057 (0.038, 0.084)	<0.001
Marital status			
Others	769 (10.76%)	Reference	
Married and cohabiting	6,376 (89.24%)	0.516 (0.438, 0.609)	<0.001
Retirement			
No	6,275 (87.82%)	Reference	
Yes	870 (12.18%)	0.315 (0.246, 0.405)	<0.001
Current smoking			
No	4,831 (67.61%)	Reference	
Yes	2,313 (32.37%)	0.773 (0.681, 0.878)	<0.001
Current drinking			
No	4,632 (64.83%)	Reference	
Yes	2,513 (35.17%)	0.728 (0.643, 0.825)	<0.001
Socializing			
No	3,340 (46.75%)	Reference	
Yes	3,805 (53.25%)	0.531 (0.472, 0.597)	<0.001
Hypertension			
No	3,741 (52.36%)	Reference	
Yes	3,404 (47.64%)	1.293 (1.152, 1.452)	<0.001
Diabetes			
No	6,081 (85.11%)	Reference	
Yes	1,064 (14.89%)	1.028 (0.875, 1.208)	0.736
Dyslipidemia			
No	6,410 (89.71%)	Reference	
Yes	735 (10.29%)	0.753 (0.614, 0.924)	0.007
Stroke			
No	6,980 (97.69%)	Reference	
Yes	165 (2.31%)	1.139 (0.786, 1.650)	0.491
Depressive symptoms			
No	4,613 (64.56%)	Reference	
Yes	2,532 (35.44%)	2.136 (1.900, 2.402)	<0.001
SBP (mmHg)	129.29 ± 21.16	1.010 (1.007, 1.013)	<0.001
DBP (mmHg)	75.52 ± 12.15	0.998 (0.993, 1.003)	0.357
BMI (kg/m^2^)	23.63 ± 3.85	0.937 (0.922, 0.953)	<0.001
FPG (mg/dL)	102.42 (94.50, 113.58)	1.000 (0.998, 1.001)	0.846
TC (mg/dL)	193.56 ± 37.68	1.000 (0.998, 1.001)	0.950
TG (mg/dL)	106.20 (75.23, 154.88)	0.999 (0.998, 1.000)	0.001
LDL-C (mg/dL)	116.60 ± 34.69	1.000 (0.998, 1.001)	0.561
HDL-C (mg/dL)	50.97 ± 15.14	1.009 (1.006, 1.013)	<0.001
hsCRP (mg/L)	1.03 (0.55, 2.14)	1.008 (1.001, 1.015)	0.017
TCBI^a^	3.09 ± 0.31	0.488 (0.401, 0.594)	<0.001

BMI, body mass index; CI, confidence interval; DBP, diastolic blood pressure; FPG, fasting plasma glucose; HDL-C, high-density lipoprotein cholesterol; hsCRP, high-sensitivity C-reactive protein; LDL-C, low-density lipoprotein cholesterol; OR, odds ratio; SBP, systolic blood pressure; TC, total cholesterol; TCBI, triglycerides, total cholesterol, and body weight index; TG, triglyceride.

a The TCBI value underwent a log transformation in univariate analysis.

### Association between TCBI and cognitive impairment

**Figure 2:**
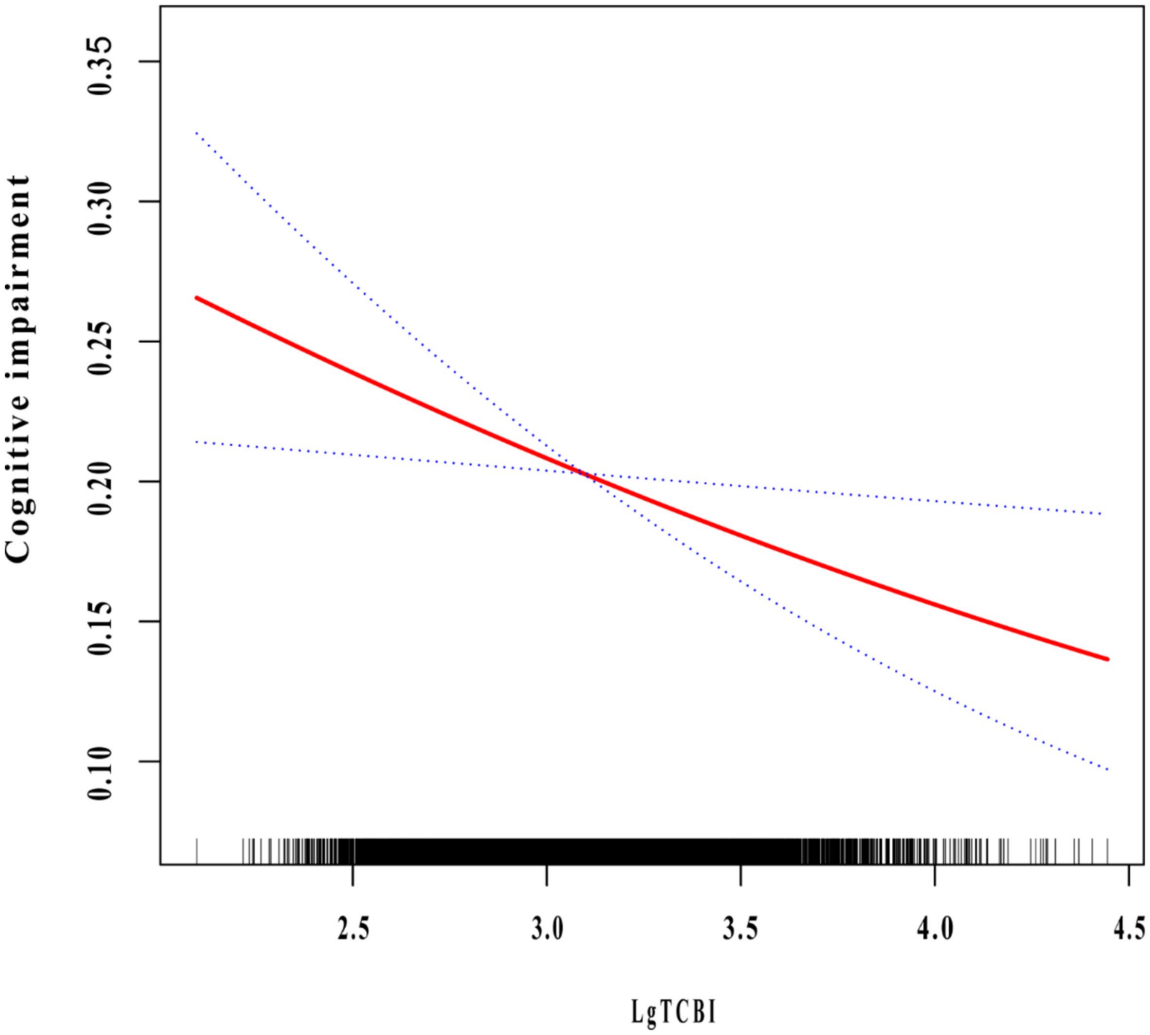
The association between TCBI and cognitive impairment. The relationship was detected after adjusting for age, sex, residence, education, marital status, retirement, current smoking, current drinking, socializing, hypertension, diabetes, dyslipidemia, stroke, depression symptoms, SBP, DBP, BMI, FPG, LDL-C, HDL-C, and hsCRP.

We conducted a multivariable logistic regression analysis to evaluate the correlation between TCBI and cognitive impairment (Table 3). After full adjustment (Model 3), the probability of cognitive impairment decreased by 29.7% for every 1 unit increase in Lg TCBI (OR = 0.703, 95% CI: 0.529–0.933; *p* = 0.015). When Lg TCBI was treated as a 4-category variable, in Model 3, the incidence of cognitive impairment in the Q2, Q3, and Q4 groups was reduced by 19.9% (OR = 0.801, 95% CI: 0.667–0.962; *p* = 0.018), 16.3% (OR = 0.837, 95% CI: 0.684–1.024; *p* = 0.084), and 22.9% (OR = 0.771, 95% CI: 0.612–0.972; *p* = 0.028) (*p* for trend = 0.043) than the Q1 group. The dose-response relationship between Lg TCBI and cognitive impairment was investigated using smooth curve fitting. The results showed that the incidence of cognitive impairment decreased as Lg TCBI levels increased (Figure 2).

**Table 3:** Association between TCBI and cognitive impairment.

TCBI^a^	OR (95% CI), *p*		
	Model 1	Model 2	Model 3
TCBI	0.488 (0.401, 0.594) <0.001	0.508 (0.412, 0.625) <0.001	0.703 (0.529, 0.933) 0.015
TCBI quartile [median (range)]			
Q1 [2.75 (<2.87)]	Reference	Reference	Reference
Q2 [2.98 (2.87–3.06)]	0.702 (0.599, 0.821) <0.001	0.698 (0.592, 0.823) <0.001	0.801 (0.667, 0.962) 0.018
Q3 [3.17 (3.07–3.28)]	0.693 (0.592, 0.812) <0.001	0.669 (0.567, 0.798) <0.001	0.837 (0.684, 1.024) 0.084
Q4 [3.45 (≥3.29)]	0.562 (0.477, 0.662) <0.001	0.573 (0.483, 0.681) <0.001	0.771 (0.612, 0.972) 0.028
*p* for trend	<0.001	<0.001	0.043

CI, confidence interval; OR, odds ratio; TCBI, triglycerides, total cholesterol, and body weight index. Model 1 was unadjusted. Model 2 was adjusted for age and sex. Model 3 was adjusted for age, sex, residence, education, marital status, retirement, current smoking, current drinking, socializing, hypertension, diabetes, dyslipidemia, stroke, depression symptoms, SBP, DBP, BMI, FPG, LDL-C, HDL-C, and hsCRP.

a The TCBI value underwent a log transformation.

### Subgroup analyses

Stratified analyses were conducted to evaluate the correlation between TCBI and cognitive impairment in the different subgroups (Table 4). Significant interactions were observed for age, current drinking, and socialization (*p* < 0.05). The negative impact of TCBI on cognitive impairment was more pronounced in participants aged ≥60 years (OR = 0.655, 95% CI: 0.438–0.979; *p* for interaction = 0.039), non-current drinking (OR = 0.643, 95% CI: 0.451–0.917; *p* for interaction = 0.015), and those engaged in socializing (OR = 0.568, 95% CI: 0.371–0.871; *p* for interaction = 0.009).

**Table 4:** The effect size of TCBI on cognitive impairment in the subgroup.

Characteristics	*N*	OR (95% CI), *p*-value	*p* for interaction
**Age (years)**			0.026
45–60	4,001	0.716 (0.480, 1.066) 0.100	
≥60	3,144	0.655 (0.438, 0.979) 0.039	
**Sex**			0.480
Male	3,623	0.529 (0.343, 0.818) 0.004	
Female	3,522	0.885 (0.602, 1.301) 0.535	
**BMI (kg/m^2^)**			0.294
<24	4,148	0.812 (0.567, 1.164) 0.257	
≥24	2,997	0.516 (0.329, 0.811) 0.004	
**Residence**			0.546
City	2,668	1.072 (0.621, 1.852) 0.802	
Rural	4,477	0.613 (0.439, 0.856) 0.004	
**Education**			0.450
Illiteracy	2,979	0.679 (0.482, 0.957) 0.027	
Primary school	1,705	0.840 (0.431, 1.639) 0.610	
Middle school	1,633	0.658 (0.255, 1.696) 0.386	
High school and above	828	0.591 (0.127, 2.752) 0.503	
**Marital status**			0.796
Married and cohabiting	6,376	0.783 (0.577, 1.062) 0.116	
Others	769	0.279 (0.123, 0.636) 0.002	
**Retirement**			0.778
No	6,275	0.694 (0.518, 0.930) 0.015	
Yes	870	0.678 (0.202, 2.268) 0.528	
**Current smoking**			0.710
No	4,831	0.768 (0.545, 1.081) 0.130	
Yes	2,313	0.556 (0.330, 0.938) 0.028	
**Current drinking**			0.034
No	4,632	0.643 (0.451, 0.917) 0.015	
Yes	2,513	0.783 (0.484, 1.266) 0.318	
**Socializing**			0.010
No	3,340	0.810 (0.552, 1.190) 0.283	
Yes	3,805	0.568 (0.371, 0.871) 0.009	
**Hypertension**			0.326
No	3,741	0.714 (0.470, 1.084) 0.114	
Yes	3,404	0.681 (0.460, 1.007) 0.054	
**Diabetes**			0.794
No	6,081	0.707 (0.511, 0.979) 0.037	
Yes	1,064	0.475 (0.249, 0.906) 0.024	
**Dyslipidemia**			0.684
No	6,410	0.700 (0.520, 0.943) 0.019	
Yes	735	0.840 (0.311, 2.267) 0.730	
**Stroke**			0.942
No	6,980	0.702 (0.526, 0.936) 0.016	
Yes	165	0.633 (0.090, 4.450) 0.646	
**Depressive symptoms**			0.648
No	4,613	0.627 (0.428, 0.918) 0.017	
Yes	2,532	0.814 (0.532, 1.248) 0.346	

CI, confidence interval; OR, odds ratio; BMI, body mass index. Above models were adjusted for age, sex, residence, education, marital status, retirement, current smoking, current drinking, socializing, hypertension, diabetes, dyslipidemia, stroke, depression symptoms, SBP, DBP, BMI, FPG, LDL-C, HDL-C, and hsCRP. In each case, the model was not adjusted for the stratification variable. The TCBI value underwent a log transformation.

### Sensitivity analyses

After excluding participants with dyslipidemia, we evaluated the correlation between TCBI and cognitive impairment by multiple logistic regression analysis (Table 5). After adjusting for age, sex, residence, education, marital status, retirement, current smoking, current drinking, socializing, hypertension, diabetes, stroke, depression symptoms, SBP, DBP, BMI, FPG, and hsCRP, the probability of cognitive impairment decreased by 32.7% for every 1 unit increase in Lg TCBI (OR = 0.673, 95% CI: 0.515–0.878; *p* = 0.004). When Lg TCBI was treated as a 4-category variable, the incidence of cognitive impairment in the Q2, Q3, and Q4 groups was reduced by 21.6% (OR = 0.784, 95% CI: 0.650–0.947; *p* = 0.011), 19.6% (OR = 0.804, 95% CI: 0.662–0.976; *p* = 0.027), and 24.3% (OR = 0.757, 95% CI: 0.610–0.940; *p* = 0.012) (*p* for trend = 0.018) than the Q1 group. The negative relationship between TCBI and cognitive impairment is robust and reliable.

**Table 5:** Association between TCBI and cognitive impairment in participants with normal blood lipids.

TCBI^a^	OR (95% CI), *p*-value
TCBI	0.673 (0.515, 0.878) 0.004
TCBI quartile [median (range)]	
Q1 [2.75 (<2.86)]	Reference
Q2 [2.96 (2.86–3.05)]	0.784 (0.650, 0.947) 0.011
Q3 [3.15 (3.06–3.25)]	0.804 (0.662, 0.976) 0.027
Q4 [3.42 (≥3.26)]	0.757 (0.610, 0.940) 0.012
*P* for trend	0.018

CI, confidence interval; OR, odds ratio; TCBI, triglycerides, total cholesterol, and body weight index. This model was adjusted for age, sex, residence, education, marital status, retirement, current smoking, current drinking, socializing, hypertension, diabetes, stroke, depression symptoms, SBP, DBP, BMI, FPG, and hsCRP.

a The TCBI value underwent a log transformation.

## Discussion

In this cross-sectional study involving 7,145 middle-aged and elderly Chinese individuals, we found a significant association between overnutrition and cognitive impairment. The results indicate that an increase in TCBI levels is related to a reduced risk of cognitive impairment, particularly among participants aged ≥60 years, non-current drinkers, and those actively engaged in social activities.

In recent years, the correlation between nutritional status and disease occurrence and prognosis has received increasing attention. Previous nutritional assessment methods, such as the PNI, COUNT, and GNRI have not been extensively used in clinical practice because of their complexity and the numerous parameters required for their calculation. In 2017, Doi et al. (13) proposed a nutritional index known as the TCBI, which only requires triglycerides, total cholesterol, and body weight for calculation. Subsequent studies demonstrated that TCBI is an effective prognostic indicator in patients with coronary heart disease (13), MCS devices (14), and heart failure (15). Shi et al. (16) also indicated that TCBI was negatively correlated with the incidence of stroke in patients with hypertension. Liu et al. (17) found that TCBI reduced the risk of SAP. However, the relationship between TCBI and cognitive impairment remains unclear. To the best of our knowledge, this study is the first to explore the correlation between TCBI and cognitive impairment.

The relationship between undernutrition and cognitive impairment is complex and not yet fully understood. A review of neurodegenerative diseases indicated that undernutrition and low BMI are associated with dementia and higher mortality rates (21). Another recent review showed that undernutrition may lead to a cognitive decline, while improved nutritional status may enhance cognitive function (7). Loda et al. (9) suggested that individuals in the early stages of cognitive impairment are susceptible to energy-protein undernutrition and micronutrient deficiencies. A cross-sectional study by He et al. (8) found that the nutritional status of patients with Alzheimer’s disease (AD) was significantly worse than that of the control group and tended to worsen with the progression of AD. Tsutsumiuchi et al. (22) discovered that most patients with post-stroke cognitive impairment (PSCI) are malnourished, which is closely linked to their prognosis. Additionally, guidelines from the European Society for Clinical Nutrition and Metabolism (ESPEN) suggest a vicious cycle between malnutrition and cognitive impairment, recommending nutritional care and support as part of dementia management (23). In our study, regardless of whether TCBI was treated as a continuous or categorical variable, the results consistently showed that higher TCBI levels reduced the risk of cognitive impairment, with smooth curve fitting further validated this negative correlation.

However, a recent review suggests that over-nutrition leads to cognitive decline by affecting insulin resistance, gut-brain axis, and neuroinflammation (24). The contrasting conclusions regarding the impact of undernutrition or overnutrition on cognition mirror debates surrounding BMI in the context of the obesity paradox or reverse epidemiology. A study by Dramé and Godaert (25) found that obesity in older adults may be associated with a lower risk of death, particularly among individuals with chronic diseases. While BMI as a measurement has limitations, as it fails to reflect differences in body fat and muscle mass. Bosello and Vanzo (26) suggested that traditional weight indicators (such as BMI) fail to accurately reflect the health status of elderly individuals, leading to an underestimation of the impact of obesity on morbidity and mortality. Similarly, current studies lack consistent methods for screening nutritional status, uniform assessment tools for cognitive function, and large-scale randomized controlled trials, hindering the establishment of a causal relationship between nutritional status and cognitive impairment. Extensive research is warranted to determine the most beneficial nutritional status for health, chronic diseases prevention, and longevity.

The mechanisms linking nutritional status and cognitive function remain unclear. Better nutritional status can provide a variety of amino acids to improve cognitive function by synthesizing neurotransmitter precursors, maintaining brain function, reducing brain inflammation, and increasing muscle protein synthesis (27). Nutrients, such as omega-3 fatty acids, B vitamins, and antioxidants counteract the pathological processes that cause cognitive impairment (28). Increased nutritional supplementation promotes the activation of neuroprotein synthesis and enhances the formation of new cortical connections and axonal sprouting, thereby facilitating cognitive recovery (29). Sufficient serum albumin can maintain colloid osmotic pressure and blood volume, ensure adequate blood supply to the central nervous system, enhance antioxidant capacity, and reduce the risk of cognitive function impairment (30). On the other hand, undernutrition can induce inflammation and oxidative stress (31), leading to brain neuronal necrosis. Nutritional deficiency may lead to synaptic dysfunction, neuronal loss, and cortical thinning, ultimately resulting in cognitive deficits (6). Moreover, undernutrition may alter the composition of gut microbiota, leading to significant changes in autoimmune and inflammatory responses, which can contribute to the deposition of amyloid-β in AD (32, 33).

This study has several limitations. First, cross-sectional study only provided a snapshot of data at a single point in time, making it difficult to determine whether changes in TCBI precede cognitive impairment or if cognitive impairment influences variations in TCBI. So it could not establish a causal relationship between TCBI and cognitive impairment. More robust longitudinal studies are needed. Second, the assessment of cognitive function lacked brain MRI parameters. Third, our study was limited to China, and ethnic differences may have affected the generalizability of the results. Finally, the confounding factors considered in this study may not encompass all potential influencing factors.

## Conclusion

TCBI is negatively correlated with cognitive impairment in the Chinese middle-aged and elderly population, particularly among participants aged ≥60, non-current drinkers, and those engaged in social activities.

## Data Availability

The datasets presented in this study can be found in online repositories. The names of the repository/repositories and accession number(s) can be found at: https://charls.pku.edu.cn.

## Footnotes

^1^ http://charls.pku.edu.cn
